# Association between Maternal Depression Symptoms across the First Eleven Years of Their Child’s Life and Subsequent Offspring Suicidal Ideation

**DOI:** 10.1371/journal.pone.0131885

**Published:** 2015-07-07

**Authors:** Gemma Hammerton, Liam Mahedy, Becky Mars, Gordon T. Harold, Anita Thapar, Stanley Zammit, Stephan Collishaw

**Affiliations:** 1 Institute of Psychological Medicine and Clinical Neurosciences, Cardiff University, Cardiff, United Kingdom; 2 Centre for Academic Mental Health, University of Bristol, Bristol, United Kingdom; 3 Andrew and Virginia Rudd Centre for Adoption Research and Practice, School of Psychology, University of Sussex, Sussex, United Kingdom; 4 International Center for Research in Human Development, Tomsk State University, Tomsk, Tomsk Oblast, Russia; University of Oxford, UNITED KINGDOM

## Abstract

Depression is common, especially in women of child-bearing age; prevalence estimates for this group range from 8% to 12%, and there is robust evidence that maternal depression is associated with mental health problems in offspring. Suicidal behaviour is a growing concern amongst young people and those exposed to maternal depression are likely to be especially at high risk. The aim of this study was to utilise a large, prospective population cohort to examine the relationship between depression symptom trajectories in mothers over the first eleven years of their child’s life and subsequent adolescent suicidal ideation. An additional aim was to test if associations were explained by maternal suicide attempt and offspring depressive disorder. Data were utilised from a population-based birth cohort: the Avon Longitudinal Study of Parents and Children. Maternal depression symptoms were assessed repeatedly from pregnancy to child age 11 years. Offspring suicidal ideation was assessed at age 16 years. Using multiple imputation, data for 10,559 families were analysed. Using latent class growth analysis, five distinct classes of maternal depression symptoms were identified (*minimal*, *mild*, *increasing*, *sub-threshold*, *chronic*-*severe*). The prevalence of past-year suicidal ideation at age 16 years was 15% (95% CI: 14-17%). Compared to offspring of mothers with *minimal* symptoms, the greatest risk of suicidal ideation was found for offspring of mothers with *chronic*-*severe* symptoms [OR 3.04 (95% CI 2.19, 4.21)], with evidence for smaller increases in risk of suicidal ideation in offspring of mothers with *sub-threshold*, *increasing* and *mild* symptoms. These associations were not fully accounted for by maternal suicide attempt or offspring depression diagnosis. Twenty-six percent of non-depressed offspring of mothers with *chronic*-*severe* depression symptoms reported suicidal ideation. Risk for suicidal ideation should be considered in young people whose mothers have a history of sustained high levels of depression symptoms, even when the offspring themselves do not have a depression diagnosis.

## Introduction

Suicidal ideation is common in adolescence and is one of the most salient risk factors for later suicide [1]. Therefore, understanding risk factors for suicidal ideation may be important for suicide prevention strategies [2]. Given that a large proportion of young people with suicidal ideation do not present to specialist services, even when their parents are known to services [3], it is crucial to identify those most at risk in community samples.

Evidence from existing community studies suggests that maternal depressive disorder is associated with an increased risk of later suicidal ideation in offspring [4,5]. Using a sample of 240 young adolescents and their mothers (the majority of whom had a history of a mood disorder), Garber et al found evidence for an association between maternal history of mood disorder and offspring suicidal symptoms one year later (d’ = 0.13), when adjusting for offspring baseline suicidal symptoms. A more recent study that assessed suicidal ideation repeatedly over a four year period in a sample of college students also found evidence that maternal history of depressive disorder, assessed retrospectively with the student, was associated with persistent suicidal ideation in offspring [5].

The majority of previous literature has examined links between a lifetime diagnosis of maternal depression and offspring suicidal ideation [4,5]. Heterogeneity in the course, timing and severity of depression that might influence risk for offspring suicidal ideation is typically not taken into account [6]. Given that depression can be episodic or persistent, focusing only on a single time point or on presence or not of a lifetime diagnosis could give a misleading impression of the level and duration of maternal depression symptoms that offspring are exposed to. Prospective longitudinal studies enable some aspects of this heterogeneity to be captured by identifying patterns of maternal depression symptoms over time. This allows severity as well as stability or change in maternal depression symptoms over time to be considered. Several studies have demonstrated the added value of using longitudinal trajectories of maternal depression symptoms to predict offspring psychopathology over an assessment at a single point in time or predefined measures of severity and chronicity [6,7], but these have thus far not considered offspring risk of suicidal ideation or behaviour.

The reasons for an association between maternal depression and offspring suicidal ideation also remain unclear. The association could be confounded by socio-demographic risk factors that are known correlates of maternal depression [8] and are also related to offspring suicide risk [9]. Alternatively, shared genetic risk factors may confound the association with variants increasing maternal depression being transmitted and also increasing risk of offspring suicidal ideation. It is also possible that the association between maternal depression and offspring suicidal ideation reflects causal processes. It is possible, for example, that maternal depression leads to offspring suicidal ideation either due to exposure to maternal suicide attempt or by increasing risk for offspring depression [10–13]. At present, however, evidence is inconclusive and although depression itself is familial, previous studies have found that the association between maternal depression and offspring suicidal ideation is not fully explained by offspring depression [5,11]. More information from unselected population cohorts is needed to better understand the degree to which risk for suicidal ideation reflects confounding by correlated socio-demographic adversity and shared familial risk, or the role of maternal suicide attempt or offspring depressive disorder. Finally, few studies have examined whether patterns of intergenerational risk transmission differ by gender. The increase in prevalence of suicidal ideation seen in the transition from childhood to adolescence is more pronounced in females [2], and there is also some evidence that familial transmission of psychopathology is stronger in parents and children of the same sex [14].

The present investigation examines the association between maternal depression symptom course over the first eleven years of their child’s life and subsequent offspring suicidal ideation at age 16 years in a large population based birth cohort. The primary hypothesis is that variation in maternal depression symptom course from pregnancy to child age 11 years will be associated with subsequent offspring suicidal ideation at age 16 years over and above potential socio-demographic and familial confounders, with greatest risk for offspring of mothers with severe and chronic depression symptoms. However, it is also expected that sub-threshold maternal depression symptoms that persist over time will be associated with increased risk of offspring suicidal ideation. The secondary hypothesis is that the associations observed will be attenuated, but not completely explained through maternal suicide attempt or a diagnosis of depression in the offspring. Analyses will also examine whether findings are similar when examining risk for offspring lifetime suicide attempt and whether patterns of associations differ by gender.

## Methods

### Sample

Data were utilised from a large UK birth cohort study; the ‘Avon Longitudinal Study of Parents and Children’ (ALSPAC). The cohort was set up to examine genetic and environmental determinants of health and development [15]. The core enrolled sample consisted of 14,541 pregnant women resident in the former county of Avon, United Kingdom, who had an expected date of delivery between 1^st^ April 1991 and 31^st^ December 1992. Of the 14,062 live births, 13,617 were singletons and were alive at one year of age. The sample is broadly representative of the UK population, however, mothers enrolled in ALSPAC were more likely to live in owner-occupied accommodation and have a car, more likely to be married and less likely to be non-white [16]. Parents and children have been followed up regularly since recruitment via questionnaire and clinic assessments. Further details on the sample characteristics and methodology have been described previously [15,16] and detailed information about ALSPAC can be found on the study website (http://www.bristol.ac.uk/alspac). For information on all available ALSPAC data see the fully searchable data dictionary (http://www.bris.ac.uk/alspac/researchers/data-access/data-dictionary).

#### Ethics statement

Written, informed consent was obtained from all mothers who entered the ALSPAC study and ethical approval for the study was obtained from the ALSPAC Ethics and Law committee (IRB00003312) and the Local Research Ethics Committees. The ethics committee specifically approved the questionnaires and the clinic testing protocols including the methods of gaining consent.

Given that ALSPAC is a longitudinal study with many contact points with participants, consent was implied for self-completion questionnaire data when postal questionnaires were returned. All questionnaires to participants were logged when sent, reminded and returned, as were requests not to send further questionnaires. For data collected at the focus clinics, verbal consent was obtained from the parents or guardians on behalf of the children and verbal assent was obtained from the children before all measures. Verbal consent was used due to the large number of assessments at each half day clinic. Additionally, many assessments were repeat measures from earlier clinics and it was considered burdensome to ask participants to supply written consent for every measure. It was ensured that all participants were clear what was involved with each assessment and were informed that they could withdraw at any time. All written consent forms are filed securely and logged electronically.

### Missing data

**Fig 1:**
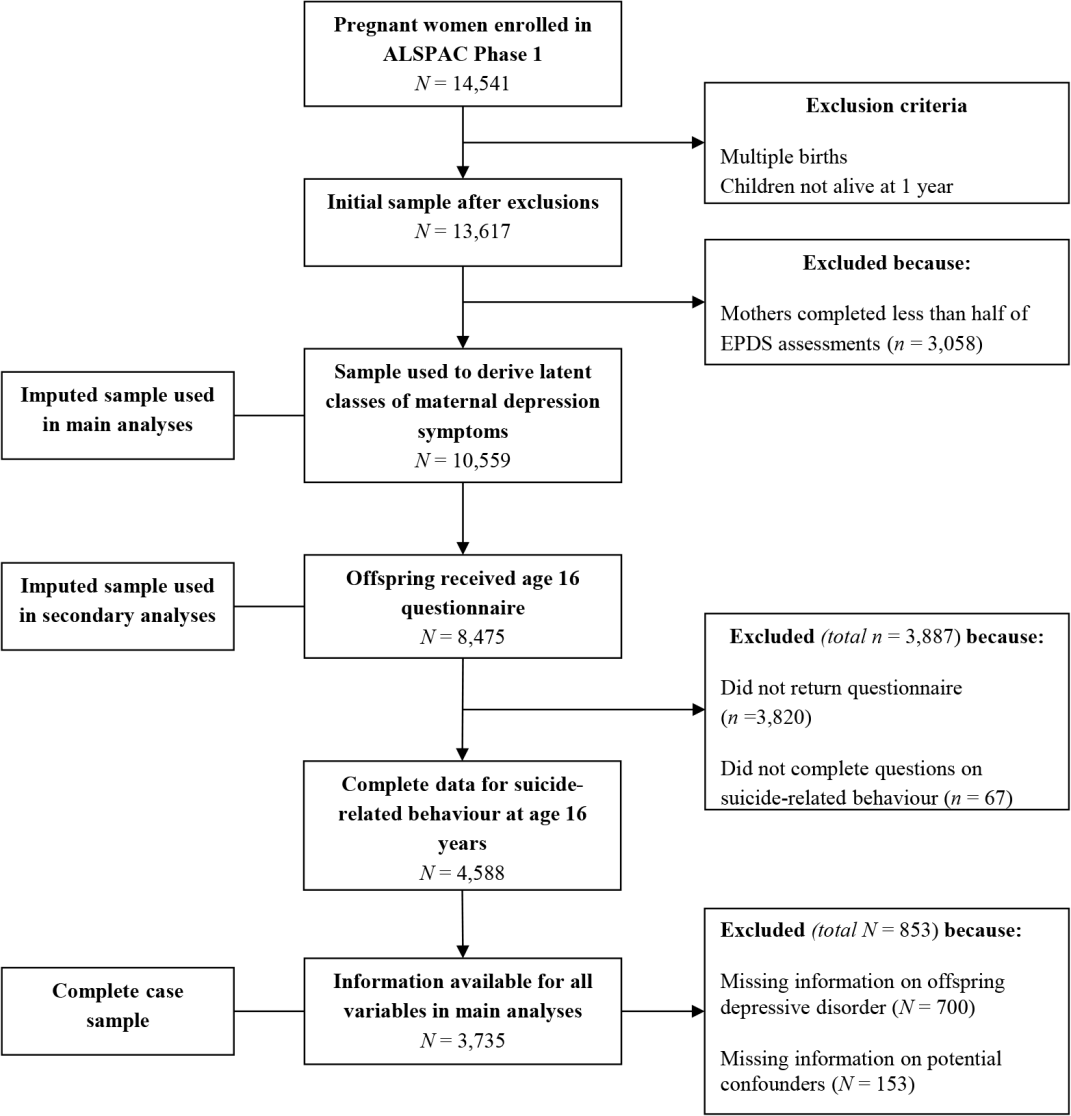
Flow chart of retention in the ‘Avon Longitudinal Study of Parents and Children’ (ALSPAC) sample.

Given that list-wise deletion of families can increase sample bias [17], methods were taken to incorporate as much data as possible and for the derivation of latent classes, missing data was handled using full information maximum likelihood (FIML) estimation [18]. The starting sample for these analyses included mothers who had information on depression symptoms from at least five time points since birth of child to age 11 years (*N* = 10,559). This was done to ensure that some data were available for each mother across the whole time period. Of the starting sample, 8,475 offspring were sent the questionnaire at age 16 years and of these 4,588 provided complete data on suicide-related behaviour (43% of starting sample; 1904 males and 2684 females; mean age: 16.7 years, standard deviation: 0.2 years). Finally, 3,735 offspring also had complete data on other covariates of interest (see Fig 1 for more details). Those missing information on outcome or covariates differed from the starting sample on a number of demographic characteristics. Mothers were younger (OR 0.92 (95% CI 0.92–0.93)) and had increased parity (OR 1.21 (95% CI 1.16–1.27)). They were also more likely to smoke in pregnancy (OR 2.21 (95% CI 1.91–2.35)), come from a lower social class (OR 1.97 (95% CI 1.81–2.14)) and be single (OR 1.73 (95% CI 1.56–1.93)) and offspring were more likely to be male (OR 0.61 (95% CI 0.56–0.66)).

Main analyses were run on those with complete data (*N* = 3,735) and on two imputed samples (*N* = 8,475; *N* = 10,559). Missing data for offspring suicide-related behaviour and depressive disorder, maternal suicide attempt and other covariates were imputed using multivariate imputation by chained equations [19] which assumes data are missing at random (MAR) i.e. given the observed data included in the imputation model, the missingness mechanism does not depend on the unobserved data [17]. As the ALSPAC sample has substantial information on socio-demographic variables that predict missingness, missing information can be assumed to be dependent on observed data. These variables were included in the imputation model to make the assumption of MAR as plausible as possible. The imputation model also included other measures that have been found to be closely associated with offspring suicide-related behaviour and depressive disorder (such as measures of offspring suicidal ideation and self-harm at other ages, depression diagnosis and symptoms at multiple time points and measures of substance abuse) and all other variables included in analyses. Using binary and multinomial logistic and linear regression models as appropriate, 80 imputed datasets were derived, each with 10 cycles of regression switching. Predictive mean matching was used when continuous variables were not normally distributed. All analyses were then run on imputed datasets by combining estimates using Rubin’s rules [17]. It has been recommended that the number of imputed datasets exceeds 100*the maximum fraction of missing information (FMI) value. FMI values were found to be no larger than 0.7, therefore imputing 80 datasets is adequate [17]. All variables with missing data used in analyses were imputed up to the maximum sample size of 10,559 (i.e. those with data on latent classes of maternal depression symptoms).

S1 Table shows demographics for those with complete data on suicide-related behaviour at age 16 years and other covariates of interest (*N* = 3,735) and the imputed sample (*N* = 10,559) in comparison to the original ALSPAC cohort that met inclusion criteria for this study (*N* = 13,617). As shown in S1 Table, the imputation procedure has corrected for biases present from selective attrition with the imputed sample being more representative of the original ALSPAC cohort than the complete case sample. Therefore, the imputed sample of 10,559 is used for all analyses hereafter; however, sensitivity checks were performed by repeating analyses using alternative approaches to dealing with missing data. First, analyses were rerun on those with complete data (*N* = 3,735) and second, data were only imputed up to the sample of offspring that were sent the questionnaire measure at age 16 years (*N* = 8,475).

### Measures

#### Maternal depression symptom trajectories

Maternal depression symptoms were assessed at 10 time points (18 weeks gestation, 32 weeks gestation, 8 weeks postnatal, 8 months postnatal, 1 year 9 months, 2 years 9 months, 5 years 1 month, 6 years 1 month, 8 years 1 month and 11 years 2 months) using the Edinburgh Postnatal Depression Scale (EPDS) [20]. The EPDS is a self-report questionnaire used to assess symptoms of depression over the past week. It includes 10 items, each rated on a 4-point scale (0–3). Examples of questions include: *I have felt sad or miserable; I have been so unhappy that I have had difficulty sleeping; I have blamed myself unnecessarily when things went wrong*. It was devised for use in the postnatal period but it has been validated for use during pregnancy and in early parenthood using standardised psychiatric interviews [21]. A cut-off at 13 has been used to predict a clinical diagnosis of depression [22]. Mothers’ scores on the EPDS at each time point correlated moderately over time (*r* = .41– .64) and internal consistencies at each time point were high (α = .85– .89).

#### Maternal suicide attempt

Maternal suicide attempt was assessed at 10 time points (from pregnancy to child age 11 years) using a self-report life events questionnaire [23] in which the mother was asked if she had attempted suicide since the previous assessment (beginning in pregnancy). All available time points were combined to create a binary ‘yes/no’ variable. Findings were robust to sensitivity analyses that examined alternative approaches to combining maternal suicide attempt across the 10 time points (i.e. only including mothers that had completed a minimum of seven assessments).

#### Mothers known to services

Mothers were considered to be known to services if they reported either seeing the doctor for their depression or taking medication for depression. Both questions were assessed at seven time points (from birth of child to child age 9 years) using a self-report questionnaire in which the mother was asked if she had seen the doctor or ‘taken pills for depression’ since the last assessment. Two binary ‘yes/no’ variables were then created by combining all available time points from birth of child to child age 3 years and then by combining all available time points from child age 3 years to 9 years. Again, findings were robust to sensitivity analyses that examined alternative approaches to combining time points.

#### Offspring suicide-related behaviour

Suicide-related behaviour at age 16 years was assessed via a self-report postal questionnaire [24]. Participants were classified as having a lifetime history of suicidal ideation if they responded positively to either of the following questions: *Have you ever found yourself wishing you were dead and away from it all*?; *Have you ever thought of killing yourself*, *even if you would not really do it*? Participants were then asked when the last time was that they felt this way. The analyses in the present investigation focus on children who reported suicidal ideation in the previous year only (78% of those who reported lifetime suicidal ideation by age 16 years) to preserve the time ordering of the analysis. History of suicidal ideation at age 11 years was assessed using the childhood interview for borderline personality disorder (CI-BPD) [25] with the question: *Have you thought about killing yourself*? This time point was solely used in later analyses to exclude those who had already experienced suicidal ideation by age 11 years to rule out the possibility of reverse causation (offspring suicidal ideation before age 11 years influencing maternal depression trajectories).

Secondary analyses investigated specific associations with lifetime history of suicide attempt by age 16 years. Participants were classified as having made a suicide attempt if they responded positively to the following question: *On any of the occasions when you have hurt yourself on purpose*, *have you ever seriously wanted to kill yourself*? Participants were also included if they reported ‘*I wanted to die’* as a reason to explain why they hurt themselves on purpose on the most recent occasion.

#### Offspring DSM-IV Major Depressive Disorder (MDD)

Offspring diagnosis of depression was assessed using the Development and Well-Being Assessment (DAWBA) [26] parent (age 7, 10 and 13 years) and child (age 15 years) versions. The DAWBA is a semi-structured interview consisting of open and closed questions about child mental health symptoms and their impact, including sections on depressive disorder, anxiety disorders, disruptive behaviour disorders, ADHD and eating disorders. DSM-IV diagnoses of MDD over the previous month were generated at each time point using a well-defined computerised algorithm that predicts the likelihood of a clinical rater assigning each child a DSM-IV diagnosis of MDD and generates diagnoses (see www.DAWBA.com for more information). A senior clinical psychiatrist reviewed the diagnoses and the DAWBA responses as part of the ALSPAC data collection process [27]. The presence of a DSM-IV diagnosis of MDD at any assessment was then calculated.

#### Potential confounders

Potential socio-demographic and familial confounding factors assessed in pregnancy were chosen based on evidence from previous literature [8,9,28,29] and associations with maternal depression symptoms and offspring suicidal ideation found in the present sample. Maternal questionnaires completed during pregnancy were used to assess housing tenure (owned vs. rented), marital status (married vs. single), maternal level of education (below O-level, O-level or above O-level; O-level, or ordinary level, is an academic qualification taken at the end of compulsory schooling which is now defunct in the UK and has been replaced with GCSE examinations), self-reported psychiatric disorder before pregnancy (yes/no; including drug addiction, alcoholism, schizophrenia, anorexia nervosa, severe depression or any other psychiatric disorder), maternal family history of depression (0, 1 or both parents) and smoking in pregnancy (smoked tobacco in either the first three months or the last two weeks of pregnancy).

### Statistical analyses

Latent class growth analysis (LCGA) [30] was used to identify qualitatively distinct patterns of depression symptoms in mothers over time from 18 weeks gestation to child age 11 years using the EPDS (as a continuous scale). In LCGA, homogenous groups of mothers are identified based on specific growth parameters including each mother’s initial level and rate of change in depression symptoms. Each mother is then given a probability of belonging to each class and these probabilities are then used to assign each mother to their most likely class. In contrast to growth mixture modeling (GMM), LCGA assumes no within class variance on the growth factors (the intercept and slope) and these are set to be zero. Given that our focus was to identify distinct groups of mothers rather than to examine within-group variability, we used the LCGA approach which helps with the clearer identification of classes and involves less computational burden than allowing the within class variance to be freely estimated [31].

From previous literature [8,28,32,33,34] we expected to find between three and six classes of maternal depression symptoms; therefore, a series of models were fitted and theoretical and statistical steps were taken to decide which model provided the best fit to the data. These included a number of fit statistics (including the sample size adjusted Bayesian information criterion (SSABIC), Lo, Mendell & Rubin likelihood ratio test (LMR-LRT) and entropy values). Using the maximum probability rule, individuals were then assigned to the class for which they had the highest probability of membership. This approach is justified when the posterior probability scores for each trajectory group are high (above at least 0.7) indicating that there is clear separation of classes [30].

To examine if variation in maternal depression symptom course was associated with subsequent offspring suicidal ideation, a logistic regression analysis was performed with maternal depression class as the exposure variable (treated as a class membership categorical variable) and past year offspring suicidal ideation at age 16 years as the outcome (model 1). In model 2, potential socio-demographic and familial confounders were adjusted for. Next maternal suicide attempt was included in analyses to examine if the association between maternal depression class and offspring suicidal ideation is explained through maternal suicide attempt (model 3). In model 4, offspring DSM-IV MDD was additionally included to examine if any association found is explained through offspring depression diagnosis. Next, analyses were rerun after excluding offspring who reported suicidal ideation at age 11 years to rule out the possibility of reverse causation (offspring suicidal ideation before age 11 years influencing maternal depression trajectories). Lastly, a logistic regression analysis was performed between classes of maternal depression symptoms (with *minimal* class as the reference group) and offspring lifetime suicide attempt by age 16 years. Analyses were conducted using Stata version 13 [35] and MPlus version 7 [36].

## Results

### Latent classes of maternal depression symptoms

Based on fit statistics, size of latent classes and parsimony, a five class model represented the best fit to the data. Model fit statistics (SSABIC (Entropy)) from 3 to 6 classes were: 505553 (0.86), 503123 (0.82), 501995 (0.80), 501003 (0.77), respectively. Lower SSABIC values reflect superior fit of a given model; however, a non-significant LMR-LRT for the 6 class model suggested that the 6 class solution did not significantly improve model fit over the 5 class solution, whereas a significant LMR-LRT for the 5 class model indicated that the 5 class model did improve model fit over the 4 class solution. The estimated posterior probability scores for each trajectory group for the five class model are presented in S2 Table. Probabilities can range from 0 to 1 with 1 representing perfect classification. Ideally, individuals’ probability of membership will approach 1 for one class with small probabilities for all other classes, indicating clear separation of classes. Average posterior probabilities for most likely latent class membership for the five class model ranged from .78 to .92 indicating relatively unambiguous classification. Findings from previous literature suggested that there could be a non-linear growth pattern to the data [7,28]; therefore a quadratic growth model was also fitted. However, as the five identified classes showed the same profile (results available on request), we chose to keep the more parsimonious model (including the linear growth parameter).

**Fig 2:**
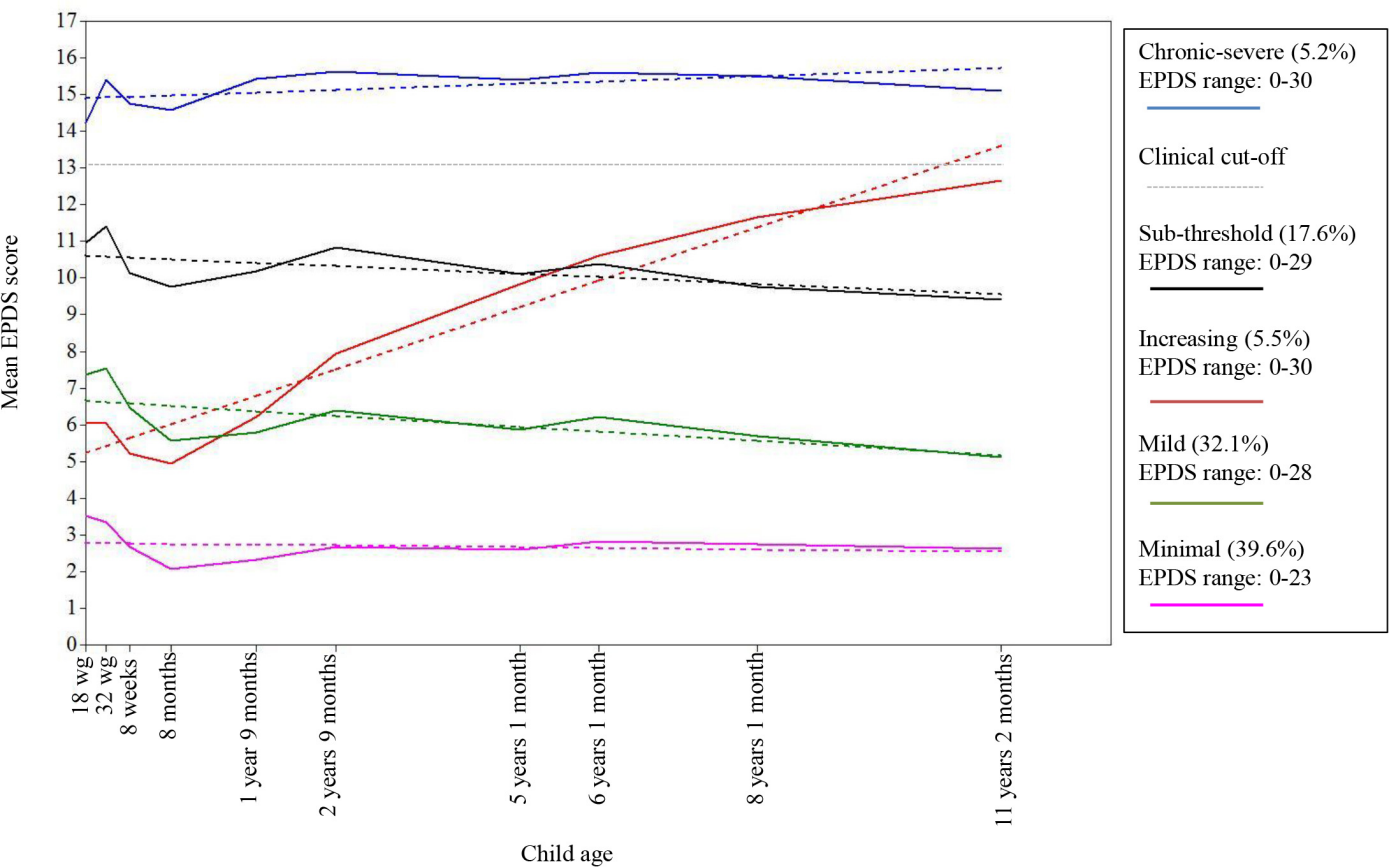
Five class model of maternal depression symptoms measured using the Edinburgh Postnatal Depression Scale (EPDS) from 18 weeks gestation to child age 11 years. Figure shows both model fitted estimated linear growth trajectories for each class (dotted line) and the observed pseudo-class trajectories for the identified classes (solid line); *N* = 10,559.

Five classes of maternal depression symptoms were identified, four showing stable levels of symptoms over time but differing in level of severity and one showing increasing symptoms. Fig 2 shows both the model fitted linear growth trajectories for each class and the observed pseudo-class trajectories for the five identified classes of maternal depression symptoms. Approximately 5% of the sample was identified as belonging to a class with high stable symptoms that were consistently above the clinical cut-off of 13 on the EPDS (*chronic-severe* class; average predicted probability of class membership: 0.93). Nearly 18% belonged to a class with sub-threshold symptoms over time, with symptom levels that were consistently just below the clinical cut-off on the EPDS and decreased very slightly over time (*sub-threshold* class; predicted probability: 0.87). Just under 6% belonged to a class with increasing symptoms over time, with symptom levels that rose to the clinical cut-off by the last time point (*increasing* class; predicted probability: 0.78). Just over 30% of the sample belonged to a class with stable mild symptoms over time (*mild* class; predicted probability: 0.81). Lastly, 40% of the sample belonged to a class with very low levels of depression symptoms over time (*minimal* class; predicted probability: 0.92). In all further analyses the *minimal* class is treated as the reference group unless otherwise stated.

### Validation of latent classes of maternal depression symptoms

Table 1 shows that the pattern of association between the classes of maternal depression symptoms and other measures used in analyses was consistent with the pattern expected. There was a stepped increase in prevalence for most measures with increasing severity of maternal depression symptom trajectories. Compared to mothers with *minimal* depression symptoms, mothers with *chronic-severe* symptoms were more likely to make a suicide attempt, live in rented accommodation, be single, smoke in pregnancy, have less education, have a psychiatric disorder before pregnancy, have a family history of depression and be known to services due to depression. Offspring of mothers with *chronic-severe* depression were also more likely to have a diagnosis of MDD. The pattern of association was similar for mothers with *mild*, *increasing* and *sub-threshold* symptoms compared to mothers with *minimal* symptoms with a few exceptions. There was no evidence that mothers with *mild* and *increasing* symptoms had less education than mothers with *minimal* symptoms. Additionally, there was no evidence that mothers with *increasing* symptoms were more likely to be single or have a family history of depression compared to mothers with *minimal* symptoms.

**Table 1:** Pattern of maternal suicide attempt, offspring DSM-IV Major Depressive Disorder (MDD), housing tenure, marital status, smoking in pregnancy, maternal level of education, maternal psychiatric disorder before pregnancy, maternal family history of depression and maternal depression-related service use by classes of maternal depression symptoms.

*%*	*Minimal(n = 4*,*177)*	*Mild(n = 3*,*384)*	*Increasing(n = 583)*	*Sub-threshold(n = 1*,*863)*	*Chronic(n = 552)*
Maternal suicide attempt	0.34	1.09***	4.12***	3.98***	10.87***
Offspring DSM-IV MDD	2.14	3.06^#^	5.87***	7.08***	14.34***
Housing tenure *(rented)*	16.05	22.77***	19.89*	27.45***	37.08***
Marital status *(single)*	16.54	22.33***	18.89	28.54***	32.37***
Smoked in pregnancy	16.86	22.90***	24.79***	29.85***	35.92***
Maternal education *(< O-level)*	24.27	25.77	23.83	31.01***	36.76***
Maternal past psychiatric disorder	4.59	10.16***	11.22***	21.45***	37.63***
Maternal family history of depression *(both parents)*	1.23	2.14**	1.95	3.40***	5.97***
Maternal service use:From birth of child to age 3 years	4.689.67	14.20***	18.14***	30.18***	55.66***
From child age 3 years to 9 years		20.67***	41.98***	39.20***	62.84***

Imputed *N* = 10,559

^#^ p < .10

*p ≤ 0.05

** p ≤ 0.01

*** p ≤ 0.001 with minimal class as the reference group

### Association between latent classes of maternal depression symptoms and offspring past year suicidal ideation at age 16 years

The number of adolescents that reported past-year suicidal ideation at the age 16 years assessment was 672/4,588 (15%; 174 males and 498 females). Of these, 81% reported specifically that they had thought about killing themselves. The number of children that reported suicidal ideation at age 11 years was 272/5,613 (5%; 144 males and 128 females). The overall prevalence was very similar in fully imputed models taking account of missing data–the estimated prevalence for past-year suicidal ideation at age 16 years was 15% (95% CI: 14–17%; 11% of males and 20% of females) and the estimated prevalence for suicidal ideation by age 11 years was 6% (95% CI: 5–6%; 6% of males and 5% of females).

**Fig 3:**
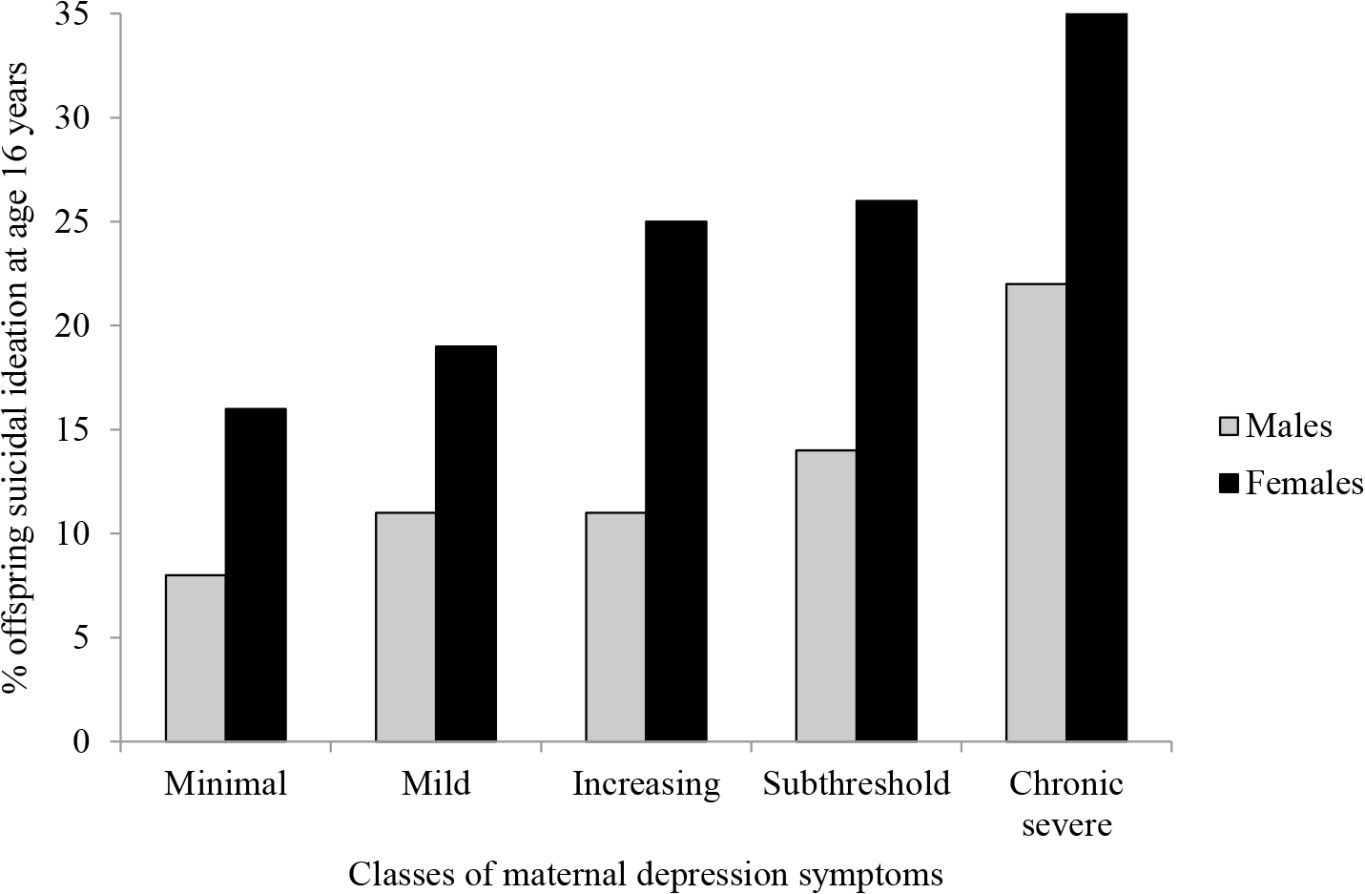
Percentage of male and female offspring with past year suicidal ideation at age 16 years for each of the classes of maternal depression symptoms. Imputed *N* = 10,559.

Fig 3 shows an increase in prevalence of offspring suicidal ideation at age 16 years with increasing severity of maternal depression symptom trajectories. The pattern is similar for males and females, although a higher percentage of females report suicidal ideation across all classes. There was no evidence of an interaction between gender and maternal depression class on offspring suicidal ideation (results available on request).

In order to examine whether the increase in prevalence of suicidal ideation at age 16 years with increasing severity of maternal depression symptoms trajectories was solely due to an increase in prevalence of offspring depressive disorder, the pattern of findings was examined in non-depressed offspring. A similar pattern of results was found when the percentage of offspring with suicidal ideation, but no previous DSM-IV diagnosis of MDD was examined [*minimal*: 11% (95% CI 10–13%)*; mild*: 14% (95% CI 12–16%); *increasing*: 16% (95% CI 12–21%); *sub-threshold*: 18% (95% CI 15–22%); *chronic-severe*: 26% (95% CI 20–32%)].

Next, a logistic regression analysis was performed between classes of maternal depression symptoms (with *minimal* class as the reference group) and offspring suicidal ideation at age 16 years. Table 2 shows evidence for increased risk of suicidal ideation in offspring of mothers from each of the depression classes in comparison to the offspring of mothers with *minimal* symptoms (model 1). These associations were attenuated only marginally when adjusted for potential confounders (model 2). Additionally, the associations were not fully explained through maternal suicide attempt (model 3) or offspring DSM-IV MDD (model 4). When excluding offspring who reported suicidal ideation at age 11 years, findings were similar (available on request). Sensitivity checks were then performed by rerunning analyses using alternative approaches to dealing with missing data. Table 3 shows associations between the classes of maternal depression symptoms and offspring suicidal ideation (after adjusting for potential confounders, maternal suicide attempt and offspring DSM-IV MDD) across the different sample sizes. Results were comparable when only imputing data for those offspring that were sent the questionnaire measure at age 16 years (*N* = 8,475; model 2). The pattern of findings was also similar when using only those with complete data on outcome and covariates, however, wider confidence intervals in complete case analysis meant that associations for the *mild* and i*ncreasing* classes dropped below significance after adjusting for all covariates (*N* = 3,735; model 3). Finally, there was no evidence that offspring gender moderated any of these associations (available on request).

**Table 2:** Logistic regression analyses showing association between each class of maternal depression symptoms in comparison to minimal class (reference group) and subsequent offspring past year suicidal ideation at age 16 years (Odds Ratios (OR) and 95% Confidence Intervals (95% CI) displayed).

	OR (95% CI)				
Maternal depression class	Model 1 (unadjusted)	Model 2 ^a^	Model 3 ^b^	Model 4 ^c^	
Minimal (*N =* 4177)	Reference group				
Mild (*N =* 3384)	1.31 (1.09, 1.59)**	1.23 (1.01, 1.49)*	1.22 (1.01, 1.49)*	1.22 (1.00, 1.48)*
Increasing (*N =* 583)Sub-threshold (*N =* 1863)	1.59 (1.16, 2.19)**1.85 (1.50, 2.27)***	1.47 (1.06, 2.04)*1.60 (1.29, 1.99)***	1.43 (1.02, 1.99)*1.57 (1.26, 1.95)***	1.37 (.97, 1.92) ^#^1.51 (1.21, 1.88)***
Chronic-severe (*N =* 552)	3.04 (2.19, 4.21)***	2.38 (1.68, 3.37)***	2.23 (1.57, 3.19)***	2.01 (1.40, 2.87)***

Imputed *N* = 10,559

^#^ p < .10

*p ≤ 0.05

** p ≤ 0.01

*** p ≤ 0.001

^a^ Adjusting for confounders assessed in pregnancy (housing tenure, marital status, maternal level of education, smoking in pregnancy, maternal family history of depression and maternal psychiatric disorder before pregnancy)

^b^ Additionally adjusting for maternal suicide attempt (from pregnancy to child age 11 years)

^c^ Additionally adjusting for DSM-IV diagnosis of MDD in offspring (assessed using the DAWBA at ages 7, 10, 13 and 15 years)

**Table 3:** Logistic regression analyses showing association between each class of maternal depression symptoms in comparison to minimal class (reference group) and subsequent offspring past year suicidal ideation at age 16 years using alternative approaches to dealing with missing data (Odds Ratios (OR) and 95% Confidence Intervals (95% CI) displayed).

	OR (95% CI) ^a^				
Maternal depression class	Model 1 (using full imputed data; *N* = 10,559) ^b^		Model 2 (imputing those that were sent Q; *N* = 8,475) ^c^		Model 3 (complete cases; *N* = 3,735) ^d^
Minimal	Reference group				
Mild	1.22 (1.00, 1.48)*	1.22 (.98, 1.50) ^#^		1.17 (.93, 1.47)	
Increasing Sub-threshold	1.37 (.97, 1.92) ^#^1.51 (1.21, 1.88)***	1.38 (.96, 1.97) ^#^1.50 (1.19, 1.90)***		1.27 (.84, 1.90)1.58 (1.20, 2.09)***	
Chronic-severe	2.01 (1.40, 2.87)***	2.08 (1.43, 3.02)***		2.03 (1.31, 3.15)**	

^#^ p < .10

*p ≤ 0.05

** p ≤ 0.01

*** p ≤ 0.001

^a^ All models adjusted for confounders assessed in pregnancy, maternal suicide attempt and DSM-IV diagnosis of MDD in offspring

^b^ Model 1 shows the fully adjusted results using the full imputed dataset

^c^ Model 2 shows the fully adjusted results using imputed data for those offspring that were sent the questionnaire at age 16 years

^d^ Model 3 shows the fully adjusted results using only those with complete data on all variables in analysis

Next, a logistic regression analysis was performed between classes of maternal depression symptoms (this time with *chronic-severe* class as the reference group) and offspring past year suicidal ideation at age 16 years. There was evidence for decreased risk of suicidal ideation in offspring of mothers from each of the depression classes in comparison to the offspring of mothers with *chronic-severe* symptoms [*minimal*: OR 0.33 (95% CI 0.24–0.46); *mild*: OR 0.43 (95% CI 0.31–0.60); *increasing*: OR 0.52 (95% CI 0.34–0.81); *sub-threshold*: OR 0.61 (95% CI 0.43–0.86)].

### Association between latent classes of maternal depression symptoms and offspring lifetime suicide attempt by age 16 years

The number of adolescents that reported lifetime suicide attempt at the age 16 years assessment was 302/4,588 (7%; 61 males and 241 females). The overall prevalence was similar in fully imputed models taking account of missing data– 8% (95% CI: 7–9%; 5% of males and 11% of females). S3 Table shows evidence for increased risk for suicide attempt in offspring of mothers with *mild*, *sub-threshold* and *chronic-severe* symptoms in comparison to the offspring of mothers with *minimal* symptoms after adjusting for potential confounders, maternal suicide attempt and offspring DSM-IV MDD.

## Discussion

In this population sample, five distinct classes of maternal depression symptoms were identified. Four classes showed stable levels of depression symptoms but differed in the level of severity and one class showed a change in severity, with increasing levels of depression symptoms over time. Variation in maternal depression symptom course was associated with subsequent offspring suicidal ideation at age 16 years, with greatest risk of suicidal ideation for offspring of mothers with *chronic-severe* depression symptoms. However, there were also smaller increases in risk for offspring of mothers with *sub*-*threshold*, *increasing* and *mild* symptoms in comparison to offspring of mothers with *minimal* symptoms. This is an important finding because more than half of this population cohort of teenagers had experienced maternal depression at these levels. Associations between maternal depression and offspring suicidal ideation were not completely explained through maternal suicide attempt or a diagnosis of depression in the offspring. Results were similar when examining associations with offspring lifetime suicide attempt.

This is one of the first studies to examine whether there is an association between variation in maternal depression symptom course over time and subsequent offspring suicidal ideation. The results extend the findings of previous longitudinal studies that have found an association between maternal depressive disorder and offspring suicidal ideation [4,5] by examining the course of maternal depression symptoms across a long time span and so taking account of the heterogeneity in the course, timing and severity of maternal depression. The identified trajectories also extend the majority of wider research on the course of maternal depression in the general population in terms of the time scale and number of assessments used. Other population-based samples that have examined trajectories of maternal depression symptoms across shorter time spans, or fewer assessments in relation to other outcomes in offspring have also identified a small class of mothers with chronic and severe depression symptoms and a number of more common classes of mothers with stable symptoms across time that differ in level of severity [7,8,28,29,32,37]. A trajectory-based approach is useful for longitudinal cohort data where repeated assessments of mental health symptoms are available [6]. The approach means that symptom levels across time rather than a single time point can be used and therefore provides a more robust measure of depression as measurement error is accounted for when deriving trajectories. Additionally, the classes that have emerged in the mothers were meaningful in that they discriminated a group of offspring at high risk of developing suicide-related behaviour.

In this study, although maternal depression trajectories were mostly stable, one group of mothers did exhibit meaningful change in symptom levels over time. Fewer studies have identified a group of mothers with increasing symptoms over time [8,28,29,37] and it could be that this group is more likely to emerge with a longer time span of assessment [28]. Additionally, the classes that emerged in the current study support findings from two recent studies that examined trajectories of maternal depression symptoms (also assessed using the EPDS) from pregnancy to approximately child age 6 years [29,37]. Both studies, one using a community sample of mothers in Brazil [29] and the other of mothers in France [37], identified a group of mothers whose depression symptoms began to rise in the child’s preschool period, with the offspring of these mothers being at similar risk for later psychiatric disorder as the offspring of mothers with depression symptoms that started high and decreased over the study [29]. In the current study, offspring of mothers with *increasing* symptoms were nearly two times more likely to have suicidal ideation at age 16 years compared to offspring of mothers with *minimal* symptoms. It should be noted however, that not only did mothers with *increasing* symptoms have the most uncertainty in group membership in the derivation of classes, but also, wider confidence intervals meant there was more uncertainty in the association with offspring suicidal ideation, especially when using only those with complete data. In addition, there was only weak evidence for increased risk of suicide attempt in offspring of mothers with *increasing* symptoms after adjusting for potential confounders.

Previous studies have provided strong evidence that both a diagnosis of depression [38,39] and maternal suicide attempt [40,41] increase suicide risk in adolescents. In this study, associations between differing levels of maternal depression and later offspring suicidal ideation were slightly attenuated when including maternal suicide attempt and offspring depression diagnosis in the analysis, especially for the *chronic-severe* class. However, there was still evidence for an association between the classes of maternal depression symptoms and offspring suicidal ideation after accounting for these potential mediators. These findings extend results from studies that have found a retrospective diagnosis of maternal depression and maternal suicide attempt have independent links with offspring suicidal ideation and behaviour [11]. Furthermore, these results provide support for the view that maternal depression, in addition to contributing to risk for a diagnosis of depression in the offspring, may also contribute to risk for suicidal ideation through other routes [5,11]. In our study, offspring of mothers with *chronic-severe* depression symptoms were at highest risk of suicidal ideation with these offspring being over two times more likely to have suicidal ideation at age 16 years compared to offspring of mothers with *minimal* symptoms. In addition, there was evidence that offspring of mothers with *chronic-severe* symptoms were also at increased risk of suicidal ideation when compared to offspring of mothers with *sub-threshold*, *increasing* and *mild* symptoms. This is unsurprising as not only are chronic and severe symptoms of depression likely to indicate higher genetic risk, but also offspring exposed to chronic symptoms are more likely to be exposed to a variety of environmental risk factors such as a negative family environment [42]. Genetic confounding was not something that we were able to account for in this study, however it is an important consideration given evidence that longitudinal stability in depression symptoms is mainly attributable to genetic factors [43]. In this study, there was no evidence that gender moderated any of the associations. These findings support previous studies that have found no evidence of gender differences when examining the association between maternal suicide attempt and offspring suicidal ideation [41], although, the findings are in contrast to evidence that the association between mother and child depression symptoms is stronger for girls compared to boys [14]. However, given that adolescent boys were more likely to drop out of the study; this may have affected our ability to detect gender differences.

Our findings need to be considered in the light of some additional limitations. Firstly, as with most cohort studies, there was selective attrition over time; however, potential bias arising from missing data was dealt with using multiple imputation, utilising a large amount of additional information to make the assumption of missing-at-random plausible [17]. Previous studies have recommended using multiple imputation to deal with potential bias arising from missing data, especially when data are thought to be missing at random (conditional on the other variables included in the model) [44]. Additionally, analyses were repeated using only those with complete data and the pattern of findings was the same except for weaker evidence of an association for the *increasing* and *mild* classes after adjusting for all covariates. This pattern of findings across imputed and complete case samples has been shown previously in studies using the same sample that have reported that the association between maternal and offspring depression may be underestimated in complete case analyses [45]. It is also important to note that approximately 3000 families were excluded from the study initially due to substantial missing data on maternal depression symptoms and other measures in analyses. Those participants excluded from the study had higher levels of socio-demographic risk factors than those that remained in, meaning that even the associations observed in the fully imputed sample may reflect conservative estimates. However, these mothers were excluded to ensure that some data were available on depression symptoms for each mother across the whole childhood period. Additionally, S1 Table showed that the imputed sample of 10,559 was representative of the original ALSPAC cohort. Second, it is important to consider the possibility of reverse causation i.e. offspring suicidal ideation having an adverse effect on maternal depression symptom course. Even though offspring suicidal ideation was assessed approximately five years after the maternal depression trajectories, some offspring may already have experienced suicidal ideation at earlier time points. However, when excluding offspring that reported suicidal ideation at age 11 years, conclusions remained unchanged suggesting that reverse causation is unlikely to explain the associations observed. Third, the importance of offspring depression as a mediator of intergenerational links may be underestimated. Although the presence of offspring DSM-IV MDD was assessed repeatedly from age 7 to age 15 years findings may not account for offspring that had an episode of depression between assessments or at age 16 years when suicidal ideation was assessed. It is also possible that concurrent sub-threshold symptoms of offspring depression at age 16 years could further explain associations. However, diagnostic measures of depression were unavailable at age 16 years and broader symptom screens typically also tap into a range of other related psychopathology, personality traits and cognitive processes. The overlap between suicidal ideation and depression symptoms in adolescents is difficult to disentangle and is something that future research should investigate more thoroughly. Fourth, we treated the derived classes of maternal depression symptoms as observed groups in analyses examining the association with offspring suicide-related behaviour. This approach means that the uncertainty in latent class membership is not taken into account and can inflate differences between classes that are not well separated. However, when the posterior probability scores for each trajectory group are high, as in the current study, this indicates clear separation of classes and provides justification for the approach that we have taken. Therefore, it is unlikely that not taking account of the uncertainty in class membership would substantially bias the findings. Finally, risk to offspring from paternal depression was not considered, and this could reflect an important confounding factor for associations between maternal depression and offspring suicide-related behaviour.

In summary, variation in maternal depression symptoms over time was associated with subsequent offspring suicidal ideation and suicide attempt, with greatest risk for offspring of mothers with *chronic-severe* symptoms. However, suicide risk should be considered in offspring, even when maternal depression symptoms are below clinical levels. Offspring of mothers with subclinical levels of depression symptoms are an important group to consider as these offspring may be less likely to be known to services as mothers may have never been diagnosed with clinical depression. In this sample, only half of mothers with *sub-threshold* symptoms over time were known to services. However, as expected, it is offspring of mothers with both chronic and severe depression symptoms that are most at risk and a priority for preventive interventions. Additionally, as 26% of non-depressed adolescents in this group reported suicidal ideation at age 16 years, this highlights the importance of enquiring about suicidal ideation in offspring of depressed mothers, even when offspring do not have a diagnosis of depression. Given that the majority of mothers from the *chronic-severe* class were already known to services, this would be an easily identified high risk group to target [3]. These results may have implications for adult mood disorder clinics, GPs and schools. The findings highlight the importance of GPs being aware of potential problems in children of mothers with chronic depression, and the need for adult services to consider risks in adolescent offspring of mothers with severe and chronic depression. Identifying barriers to effective communication between adult and child services will be important. The results also highlight the importance of educating schools in being able to identify children at high suicide risk, and knowing the appropriate course of action to take, given that, for some children at risk, it is possible that neither parent nor child will already be known to services. Future research is needed to replicate these findings and to examine other potential mechanisms that may explain these associations.

## Supporting Information

S1 Table Demographics of the two main samples used in analyses and the original cohort that met inclusion criteria. (DOCX)

S2 Table Average posterior probability scores for most likely latent class membership (row) by latent class (column) for the five class model. Bold represents average posterior class probability for trajectory membership.
(DOCX)

S3 Table Logistic regression analysis showing associations between each class of maternal depression symptoms in comparison to minimal class (reference group) and offspring lifetime suicidal attempt by age 16 years (Odds Ratios (OR) and 95% Confidence Intervals (95% CI) displayed). Imputed N = 10,559.
(DOCX)
