# Correction: Association between Maternal Depression Symptoms across the First Eleven Years of Their Child's Life and Subsequent Offspring Suicidal Ideation

**DOI:** 10.1371/journal.pone.0136367

**Published:** 2015-08-14

**Authors:** Gemma Hammerton, Liam Mahedy, Becky Mars, Gordon T. Harold, Anita Thapar, Stanley Zammit, Stephan Collishaw

There are errors in Tables 2 and 3. The correct tables are here.

**Table 2:** Logistic regression analyses showing association between each class of maternal depression symptoms in comparison to minimal class (reference group) and subsequent offspring past year suicidal ideation at age 16 years (Odds Ratios (OR) and 95% Confidence Intervals (95% CI) displayed).

	OR (95% CI)			
Maternal depression class	Model 1 (unadjusted)	Model 2 ^a^	Model 3 ^b^	Model 4 ^c^
Minimal (*N* = 4177)	Reference group			
Mild (*N* = 3384)	1.31 (1.09, 1.59)**	1.23 (1.01, 1.49)*	1.22 (1.01, 1.49)*	1.22 (1.00, 1.48)*
Increasing (*N* = 583)	1.59 (1.16, 2.19)**	1.47 (1.06, 2.04)*	1.43 (1.02, 1.99)*	1.37 (.97, 1.92) ^#^
Sub-threshold (*N* = 1863)	1.85 (1.50, 2.27)***	1.60 (1.29, 1.99)***	1.57 (1.26, 1.95)***	1.51 (1.21, 1.88)***
Chronic-severe (*N* = 552)	3.04 (2.19, 4.21)***	2.38 (1.68, 3.37)***	2.23 (1.57, 3.19)***	2.01 (1.40, 2.87)***

Imputed *N* = 10,559;

^#^ p<.10;

*p ≤ 0.05;

** p ≤ 0.01;

*** p ≤ 0.001

^a^ Adjusting for confounders assessed in pregnancy (housing tenure, marital status, maternal level of education, smoking in pregnancy, maternal family history of depression and maternal psychiatric disorder before pregnancy)

^b^ Additionally adjusting for maternal suicide attempt (from pregnancy to child age 11 years)

^c^ Additionally adjusting for DSM-IV diagnosis of MDD in offspring (assessed using the DAWBA at ages 7, 10, 13 and 15 years)

**Table 3:** Logistic regression analyses showing association between each class of maternal depression symptoms in comparison to minimal class (reference group) and subsequent offspring past year suicidal ideation at age 16 years using alternative approaches to dealing with missing data (Odds Ratios (OR) and 95% Confidence Intervals (95% CI) displayed).

	OR (95% CI) ^a^		
Maternal depression class	Model 1 (using full imputed data; *N* = 10,559) ^b^	Model 2 (imputing those that were sent Q; *N* = 8,475) ^c^	Model 3 (complete cases; *N* = 3,735) ^d^
Minimal	Reference group		
Mild	1.22 (1.00, 1.48)*	1.22 (.98, 1.50) ^#^	1.17 (.93, 1.47)
Increasing	1.37 (.97, 1.92) ^#^	1.38 (.96, 1.97) ^#^	1.27 (.84, 1.90)
Sub-threshold	1.51 (1.21, 1.88)***	1.50 (1.19, 1.90)***	1.58 (1.20, 2.09)***
Chronic-severe	2.01 (1.40, 2.87)***	2.08 (1.43, 3.02)***	2.03 (1.31, 3.15)**

^#^ p<.10;

*p ≤ 0.05;

** p ≤ 0.01;

*** p ≤ 0.001

^a^ All models adjusted for confounders assessed in pregnancy, maternal suicide attempt and DSM-IV diagnosis of MDD in offspring

^b^ Model 1 shows the fully adjusted results using the full imputed dataset

^c^ Model 2 shows the fully adjusted results using imputed data for those offspring that were sent the questionnaire at age 16 years

^d^ Model 3 shows the fully adjusted results using only those with complete data on all variables in analysis
