# Supplementary material for: Association between Maternal Depression Symptoms across the First Eleven Years of Their Child’s Life and Subsequent Offspring Suicidal Ideation
Source: PLoS One. 2015 Jul 7;10(7):e0131885. doi: 10.1371/journal.pone.0131885 (PMC4495034; doi:10.1371/journal.pone.0131885)
Supplement: S1 Table — (DOCX) [file pone.0131885.s001.docx]

**S1 Table. Demographics of the two main samples used in analyses and the original cohort that met inclusion criteria.**

| Sample demographics assessed during pregnancy | Complete cases ^a^  (N = 3735) | Imputed sample ^b^  (N = 10559) | Initial cohort ^c^  (N ≤ 13617) |
| --- | --- | --- | --- |
| Female offspring (%) | 56.4 | 48.4 | 48.4 |
| Smoked in pregnancy (%) | 14.7 | 22.5 | 25.8 |
| Housing tenure (% rented) | 12.7 | 21.5 | 26.7 |
| Marital status (% single) | 15.7 | 21.5 | 23.5 |
| Maternal education (% < O-level) | 16.1 | 26.6 | 26.7 |
| Maternal depression (mean EPDS score at 32 weeks gestation) | 6.16 | 6.90 | 7.05 |

^a^ Sample with complete data on offspring suicide-related behavior at age 16 years, offspring depressive disorder, maternal suicide attempt and potential confounders;

^b^ Sample with imputed data on offspring suicide-related behaviour, offspring depressive disorder, maternal suicide attempt and potential confounders to bring sample size up to all those that have information on latent classes of maternal depression symptoms;

^c^ Original ALSPAC cohort that the met inclusion criteria for this study;

Additional missing data on demographics: smoked in pregnancy missing for 792/13617; housing tenure missing for 914/13617; marital status missing for 858/13617; maternal education missing for1515 /13617; maternal Edinburgh Postnatal Depression Scale (EPDS) missing for 1895/13617
