# Supplementary material for: Association between Maternal Depression Symptoms across the First Eleven Years of Their Child’s Life and Subsequent Offspring Suicidal Ideation
Source: PLoS One. 2015 Jul 7;10(7):e0131885. doi: 10.1371/journal.pone.0131885 (PMC4495034; doi:10.1371/journal.pone.0131885)
Supplement: S2 Table — Bold represents average posterior class probability for trajectory membership. (DOCX) [file pone.0131885.s002.docx]

**S2 Table. Average posterior probability scores for most likely latent class membership (row) by latent class (column) for the five class model.** Bold represents average posterior class probability for trajectory membership.

| Most likely latent class membership | Latent class | | | | |
| --- | --- | --- | --- | --- | --- |
|  | Minimal | Mild | Increasing | Sub-threshold | Chronic-severe |
| Minimal | **.919** | .075 | .007 | .000 | .000 |
| Mild | .083 | **.814** | .058 | .046 | .000 |
| Increasing | .015 | .140 | **.784** | .061 | .000 |
| Sub-threshold | .000 | .071 | .035 | **.867** | .027 |
| Chronic-severe | .000 | .000 | .001 | .074 | **.925** |
