# Supplementary material for: Association between Maternal Depression Symptoms across the First Eleven Years of Their Child’s Life and Subsequent Offspring Suicidal Ideation
Source: PLoS One. 2015 Jul 7;10(7):e0131885. doi: 10.1371/journal.pone.0131885 (PMC4495034; doi:10.1371/journal.pone.0131885)
Supplement: S3 Table — Imputed N = 10,559. (DOCX) [file pone.0131885.s003.docx]

**S3 Table. Logistic regression analysis showing associations between each class of maternal depression symptoms in comparison to minimal class (reference group) and offspring lifetime suicidal attempt by age 16 years (Odds Ratios (OR) and 95% Confidence Intervals (95% CI) displayed).**

|  | OR (95% CI) | | | |  |  |  |  |
| --- | --- | --- | --- | --- | --- | --- | --- | --- |
| Maternal depression class | Model 1 (unadjusted) | Model 2 ^a^ | Model 3 ^b^ | Model 4 ^c^ | |  |  |  |
| Minimal (*N =* 4177) | Reference group | | | |  | |  |  |
| Mild (*N =* 3384) | 1.50 (1.14, 1.96)** | 1.35 (1.02, 1.78)* | 1.34 (1.01, 1.76) | 1.33 (1.01, 1.76)* | |  |  |  |
| Increasing (*N =* 583)  Sub-threshold (*N =* 1863) | 1.80 (1.12, 2.87)*  2.55 (1.92, 3.38)*** | 1.60 (.99, 2.58) ^#^  2.05 (1.53, 2.76)*** | 1.51 (.93, 2.46) ^#^  1.97 (1.46, 2.66)*** | 1.41 (.86, 2.31)  1.85 (1.37, 2.50)*** | |  |  |  |
| Chronic-severe (*N =* 552) | 3.79 (2.48, 5.78)*** | 2.64 (1.71, 4.08)*** | 2.35 (1.51, 3.67)*** | 1.97 (1.25, 3.12)** | |  |  |  |

Imputed N = 10,559; ^#^ p<.10; *p ≤ 0.05; ** p ≤ 0.01; *** p ≤ 0.001

^a^ Adjusting for confounders assessed in pregnancy (housing tenure, marital status, maternal level of education, smoking in pregnancy, maternal family history of depression and maternal psychiatric disorder before pregnancy)

^b^ Additionally adjusting for maternal suicide attempt (from pregnancy to child age 11 years)

^c^ Additionally adjusting for DSM-IV diagnosis of MDD in offspring (assessed using the DAWBA at ages 7, 10, 13 and 15 years)
